# Genome-wide CRISPR screens reveal synthetic lethal interaction between *CREBBP* and *EP300* in diffuse large B-cell lymphoma

**DOI:** 10.1038/s41419-021-03695-8

**Published:** 2021-04-28

**Authors:** Man Nie, Likun Du, Weicheng Ren, Julia Joung, Xiaofei Ye, Xi Shi, Sibel Ciftci, Dongbing Liu, Kui Wu, Feng Zhang, Qiang Pan-Hammarström

**Affiliations:** 1 Department of Biosciences and Nutrition, Karolinska Institutet, Stockholm, Sweden; 2 Department of Medical Oncology, Sun Yat-Sen University Cancer Center, State Key Laboratory of Oncology in South China, Collaborative Innovation Center for Cancer Medicine, Guangzhou, China; 3 Broad Institute of MIT and Harvard, Cambridge, MA USA; 4 McGovern Institute for Brain Research, Department of Brain and Cognitive Sciences, and Department of Biological Engineering, Massachusetts Institute of Technology, Cambridge, MA USA; 5 Howard Hughes Medical Institute, Cambridge, MA 02139 USA; 6 BGI-Shenzhen, Shenzhen, 518083 China; 7 Guangdong Provincial Key Laboratory of Human Disease Genomics, Shenzhen Key Laboratory of Genomics, Shenzhen, China

**Keywords:** Cancer screening, Cancer therapy

## Abstract

Diffuse large B-cell lymphoma (DLBCL) is the most common type of aggressive lymphoid malignancy and a highly heterogeneous disease. In this study, we performed whole-genome and transcriptome sequencing, and a genome-wide CRISPR-Cas9-knockout screen to study an activated B-cell-like DLBCL cell line (RC-K8). We identified a distinct pattern of genetic essentialities in RC-K8, including a dependency on *CREBBP* and *MDM2*. The dependency on *CREBBP* is associated with a balanced translocation involving *EP300*, which results in a truncated form of the protein that lacks the critical histone acetyltransferase (HAT) domain. The synthetic lethal interaction between *CREBBP* and *EP300* genes, two frequently mutated epigenetic modulators in B-cell lymphoma, was further validated in the previously published CRISPR-Cas9 screens and inhibitor assays. Our study suggests that integration of the unbiased functional screen results with genomic and transcriptomic data can identify both common and unique druggable vulnerabilities in DLBCL and histone acetyltransferases inhibition could be a therapeutic option for *CREBBP* or *EP300* mutated cases.

## Introduction

Diffuse large B-cell lymphoma (DLBCL) is one of the most common types of aggressive lymphoid malignancy. With the current standard immunochemotherapy, ~30–40% of DLBCL patients still suffer from refractory disease or relapse^1,2^. Based on transcriptional profiles, two major subtypes of DLBCL have been defined: germinal centre B-cell like (GCB) and activated B-cell like (ABC)^1^. Large-scale genome sequencing has further enabled the identification of several molecular subtypes of DLBCL based on genetic alterations affecting the proto-oncogenes *BCL2/-6* and *MYC*, epigenetic modifiers and regulators in the B-cell receptor (BCR), nuclear factor-κB (NF-κB), NOTCH and p53 signalling pathways^3,4^. The ABC subtype and selected molecular subtypes (C3 and C5 in Chapuy et al.^3^; MCD and N1 in Schmitz et al.^4^; cluster with *NOTCH1* mutations in Lacy et al.^5^) have been suggested to be associated with a poor prognosis. In addition, a new probabilistic classification tool, named LymphGen, was created to identify the genetic subgroups of DLBCL biopsy with therapeutic implications^6^. Recently, we have shown that hepatitis B virus (HBV)-related DLBCLs are associated with unique genetic and clinical features, as well as shorter overall patient survival, and may be considered a distinct subtype^7^. DLBCL is thus a highly heterogeneous disease and identification of genetic vulnerabilities that are specific to a subtype or subgroup of patients will aid in the development of novel targeted therapeutic strategies and improve clinical outcome.

One promising approach to systematically identify such genetic vulnerabilities is through genome-wide CRISPR-Cas9 screening^8^, which has been used in a range of contexts to discover genetic dependencies and vulnerabilities in cancer cells^9–11^. In the context of B-cell malignancy, CRISPR-Cas9 screens have already provided valuable results for understanding the mechanisms of tumorigenesis^12,13^ as well as discovery of drugs that might enhance tumour antigen presentation to T cells^14^.

Dysregulation of epigenetic modulators is frequently observed in lymphomas. *EZH2*, *KMT2D*, *MEF2B*, genes encoding linker histone H1 proteins, as well as *CREBBP*, the histone acetyltransferase (HAT) encoding gene and its paralogue *EP300*, are among the most frequently mutated genes in B-cell lymphomas^15–17^. Both *CREBBP* and *EP300* are large multidomain proteins that, in addition to their catalytic HAT domain, contain bromodomains (BRDs) that bind acetylated histones and are required for chromatin binding^18^. Despite the sequence homology and functional similarities between *CREBBP* and *EP300*^19^, monoallelic germline lesions in either gene may cause a severe phenotype, i.e., Rubinstein–Taybi syndrome^20^. In B-cell lymphomas, heterozygous somatic mutations in either gene may result in haploinsufficiency and these mutations have been described as mutually exclusive in most cases^17^. Genetic alterations in *CREBBP* are, however, more frequently observed in these disorders than deficiency of *EP300*. Functional screening using a small interfering RNA library suggested that *EP300* is a specific synthetic lethal gene in *CREBBP*-deficient lung cancer cells^21^. A recent study further showed that the synthetic lethal interaction between *CREBBP* and *EP300* was obtained in both normal germinal centre B cells and *CREBBP*-mutant DLBCL cells, raising the possibility of targeting *EP300* in the treatment of *CREBBP*-mutated tumours^22^.

In this study, we performed whole-genome and transcriptome sequencing to characterize an ABC-like DLBCL cell line (RC-K8) established from peritoneal effusions of a patient with terminal, refractory-stage disease^23^. We also used an unbiased, genome-wide CRISPR-Cas9 loss-of-function screening approach to study genetic dependencies in this cell line. For comparison, we have furthermore evaluated the published CRISPR-Cas9 screens from a pan-cancer study and sets of DLBCL cell lines. We identified a distinct pattern of genetic essentialities in RC-K8, including a specific dependency on *CREBBP* in the context of *EP300* deficiency. We also observed the essentiality of *EP300* in several DLBCL cell lines harbouring mutation or copy number loss in *CREBBP*. Our results confirmed and extended the previous finding on synthetic lethal interaction between the two important epigenetic modulators, and suggest that the dependency of the remaining HAT function is a druggable vulnerability in *CREBBP-* or *EP300-*deficient DLBCLs.

## Results

### Characterization of the genome and transcriptome of RC-K8 cells

**Fig. 1:**
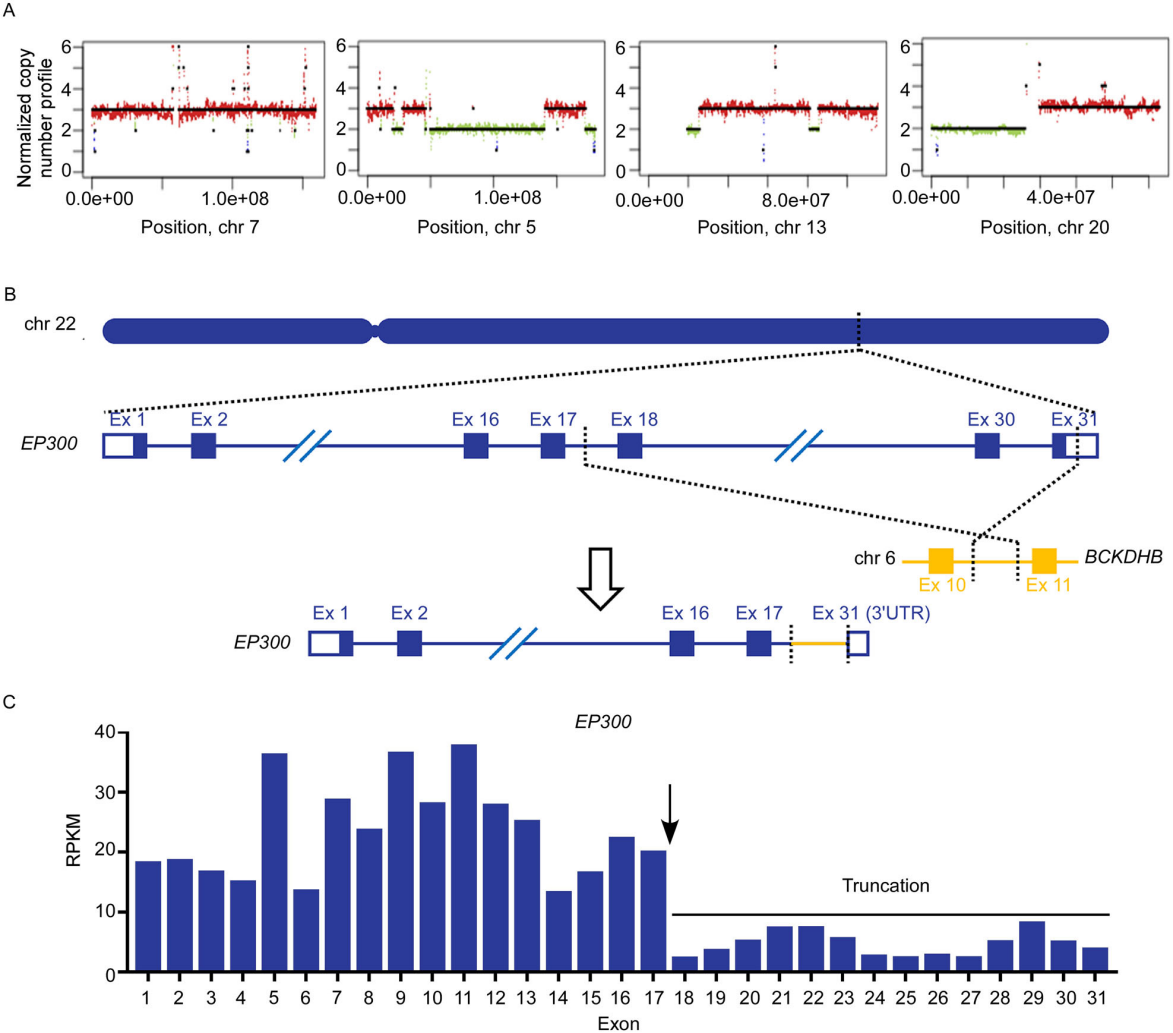
Identification of *EP300* translocation in the RC-K8 cell line by WGS and RNA-seq. **A** Whole chromosome or large partial chromosome gains of chromosomes 7, 5, 13 and 20 were observed in the RC-K8 cell line. **B** Balanced translocation identified by WGS, involving *EP300* on chromosomal 22 and *BCKDHB* on chromosomal 6, which resulted in a C-terminal truncated form of the protein. **C** Reads per exon kilobase per million (RPKM) values-based expression analysis of *EP300* in RC-K8 by RNA-seq. The expression of the truncated *EP300* gene (exons 1–17) was dominant as compared to the full-length gene.

RC-K8 cell line was established from a patient with lymphoma, described at the time as histiocytic lymphoma (terminal and refractory stage)^24^, and later was assigned to the GCB subtype of DLBCL^25^. However, more recent studies on this cell line suggested a constitutive NF-κB signalling, a feature that is usually associated with the ABC subtype^26^. Cytogenetic analysis and fluorescence in situ hybridization analysis have identified the karyotype of this cell line and a translocation involving the *BCL6* gene^27,28^. To further characterize the RC-K8 cell line, we performed whole-genome and transcriptome sequencing (RNA sequencing (RNA-seq)) on DNA and RNA samples derived from this cell line. Based on the distribution of genomic sequence coverage, we discovered trisomy of chromosome 7 and gain of part of chromosomes 5, 13 and 20 (Fig. 1A and Supplementary Fig. S1), and estimated copy number gains of *MYC* and *NOTCH1*, as well as amplification of *REL* (Supplementary Table S1). In addition, RC-K8 cells were estimated to have copy number losses of *CD70* and *UBE2A*, and two cohesion-related genes, *STAG2* and *SMC1A* (Supplementary Table S1). We further identified 384 genes with nonsynonymous mutations (*n* = 436), including genes involved in DNA damage response and repair (*RAD21*, *TP63*, *TP73*, and *XRCC6*), BCR/NF-κB signalling (*TNFAIP3* and *NFKBIA*), and transcription factors important for B-cell development (*IKZF1* and *IKZF3*) (Supplementary Table S2). Sequencing data showed that the RC-K8 cell line harbours a largely normal *TP53* gene with a benign P72R polymorphism but with a relatively high expression level of its negative regulator *MDM2* (fragments per kilobase of transcript per million mapped reads: 46.55), as estimated by RNA-seq. Through whole-genome sequencing (WGS), we also identified and mapped structural variants to base pair resolution including translocations involving *IGH* and *BCL6* (with different translocation partners), as well as a balanced translocation between chromosomes 22 and 6 in RC-K8 (Fig. 1B), which resulted in a C-terminal truncation of *EP300* that has been previously reported as EP300ΔC1047^23,29^. RNA-seq analysis further demonstrated dominant allelic expression of the truncated form of *EP300* (Fig. 1C), which is encoded by exons 1–17 of *EP300* and fused to 25 amino acids encoded by intronic sequences of the *BCKDHB* gene on chromosome 6, resulting in the loss of two critical domains of this protein, i.e., the BRD and HAT domains^23^. Finally, based on the gene expression data, RC-K8 indeed showed an ABC, rather than GCB gene expression signature (Supplementary Fig. S2)^12^. In summary, by sequencing the genome and transcriptome of RC-K8, we have characterized this cell line as an ABC-like DLBCL line, with unique genetic alterations, including a translocation that resulted in a truncation of *EP300*.

### Genome-scale CRISPR-Cas9 knockout screening reveals essential genes and pathways in RC-K8 cells

To investigate the cancer dependencies related to the genetic background of the RC-K8 cell line, we performed a functional genetic screen by introducing the genome-wide pooled CRISPR-Cas9 knockout library (detailed in “Methods”). A Cas9-expressing RC-K8 cell line (RC-K8-Cas9) was first generated and subsequently transduced by the pooled lentiCRISPRv2 library, which targets 19,050 human genes with more than 100,000 unique single-guide RNAs (sgRNAs). By deep sequencing the amplified sgRNA library, we identified depleted guide RNAs (gRNAs) caused by dropout of cells bearing related genetic perturbations, which reflects the importance of the targeted genes for cell growth or proliferation.

**Fig. 2:**
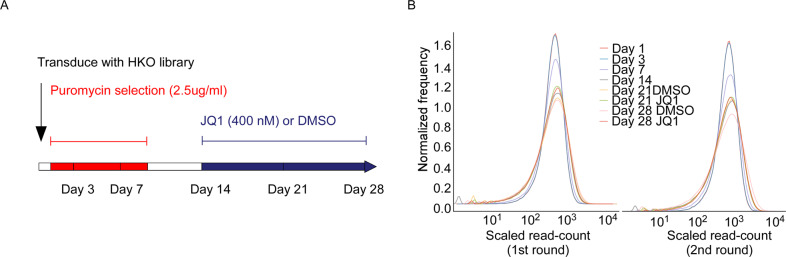
Design of the CRISPR-Cas9 loss-of-function screen in the RC-K8 cell line. **A** Time points and drug treatments. The lentiCRISPR GeCKOv2 library was transduced at day zero. Puromycin selection for successful transduction was applied between day 1 and day 10. Drug treatments (JQ1 or DMSO vehicle) were applied after midpoint day 14. Cells were collected at six time points for sequencing, including day 1 as the baseline. **B** Distribution of read counts of guide sequences (rescaled and centred by the median of 1000). Compared to the baseline and the later time points, the distribution at day 7 is distinctive and has been used for detecting essential genes that caused rapid depletion.

**Fig. 3:**
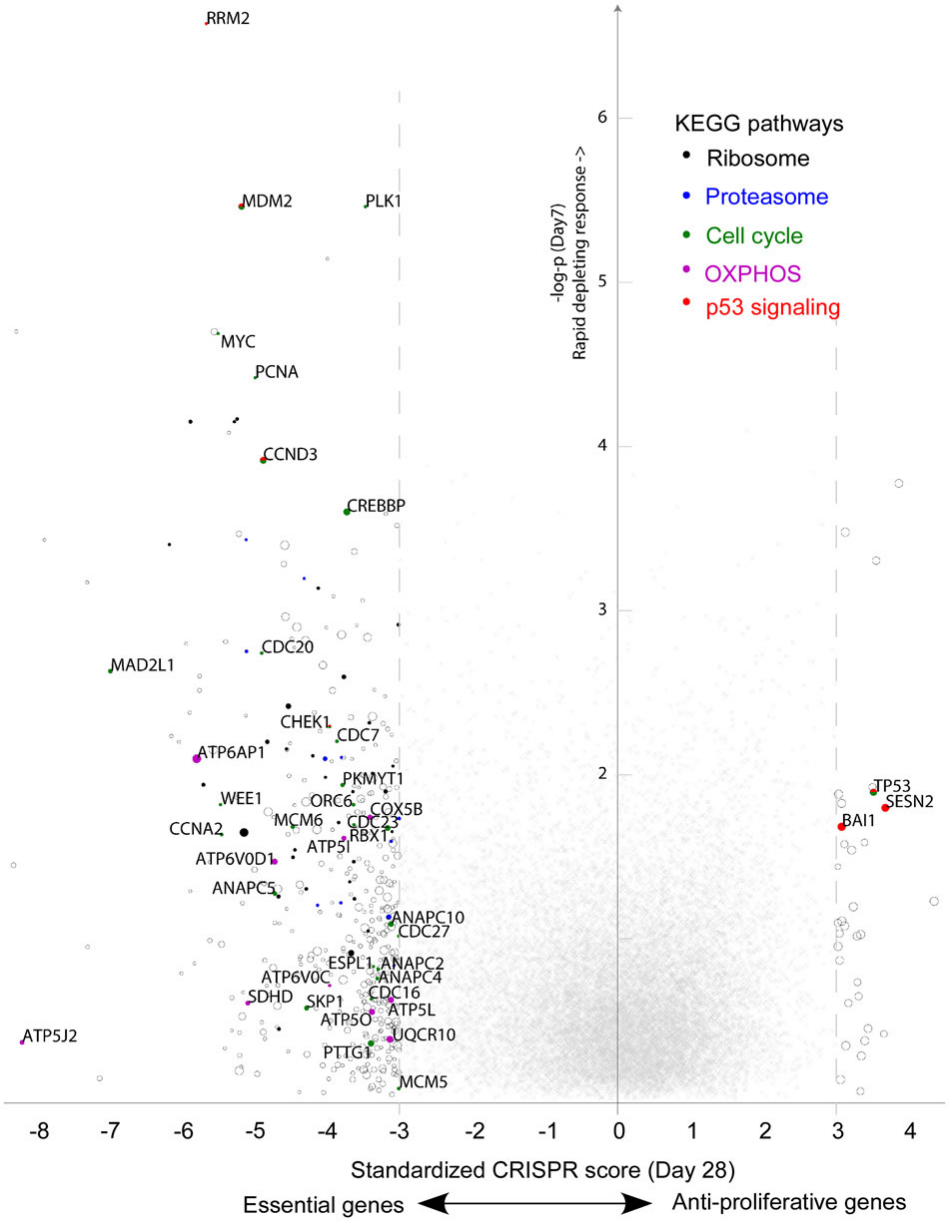
Identification of essential genes in RC-K8 based on genome-scale CRISPR-Cas9 knockout screening. *y*-axis: significance of rapid responses to genetic perturbations observed at day 7; *x*-axis: standardized (*z*-transformed) CRISPR score reflecting overall effects at day 28. Circles: genes with overall scores < −3.0 (essential genes) or >3.0 (anti-proliferative genes); circle sizes are scaled by the average CERES score reflecting general essentiality in cancer cells, where smaller circles indicate cell-specific dependencies, such as *MDM2*, *CREBBP* and *CCND3*. Genes involved in the five indicated KEGG pathways are filled with colours.

We then applied the optimized MAGeCK-based scoring system (detailed in “Methods”) to estimate the relative effects of gene knockouts during the 28-day time course of the CRISPR screen on RC-K8 cells (Fig. 2A). Based on both the read-count distribution and the standardized CRISPR scores, we observed no significant depletion at day 3 after transduction (Fig. 2B). In contrast, at day 7, we started to observe a rapid depletion of cells bearing knockouts of core fitness genes related to ribosome, spliceosome, proteasome and cell cycle regulation (Fig. 3)^9,10,30,31^. At day 7, the top candidates for core fitness genes also included cell-type specific essentialities (Supplementary Table S1). Several DLBCL essential genes, as estimated from the previous two screens^12,13^, were detected early at day 7, including *FOXO1*, *IRF4* and *SF3B1*, whereas some appeared later than day 14, e.g., *TAF1*, *MLL2*, *RHOA* and *YY1* (Supplementary Table S1). Specifically, for RC-K8 cells, which carry a largely normal *TP53* gene locus, *MDM2* was one of the most significant essential genes detected at day 7, demonstrating its critical role in suppressing TP53-induced cell death (Fig. 3 and Supplementary Table S1). When re-analysing the CERES data set (CRISPR screens on 341 cancer cell lines)^32^, we indeed observed that cancer cell lines with wild-type *TP53* in general are more dependent on *MDM2* and *MDM4* (Supplementary Fig. S3). *CCND3*, which is associated with both TP53 signalling and cell cycle regulation pathways, exhibited essentiality and specificity to the cell line (Fig. 3). Similarly, *CREBBP* was also identified at day 7 as one of the top hits, showing its essentiality in the context of *EP300* loss-of-function (Fig. 3).

On the axis of positive selection, the effects of knocking out growth-suppressing genes were generally accumulative. We observed a significant anti-proliferative effect of the classical tumour suppressor *TP53* and several associated regulators/effectors, such as *SESN2* and *BAI1* (Fig. 3). Knockouts of some “cancer drivers” of DLBCL, including *BCL2*, *BCL6*, *NFKBIA* and *CD70*, showed only modest promotional effects on cell proliferation (Supplementary Table S1).

We validated several top-ranking genes (essential genes *MDM2* and *CREBBP*, tumour suppressor gene *TP53*) from genome-wide screening by single-knockout experiments (Supplementary Fig. S4A–D) and further confirmed the rapid and complete responsiveness to *MDM2* inhibition by nutlin-3 (Supplementary Fig. S4E).

### Late time-point depletion identifies different essential pathways in RC-K8

**Fig. 4:**
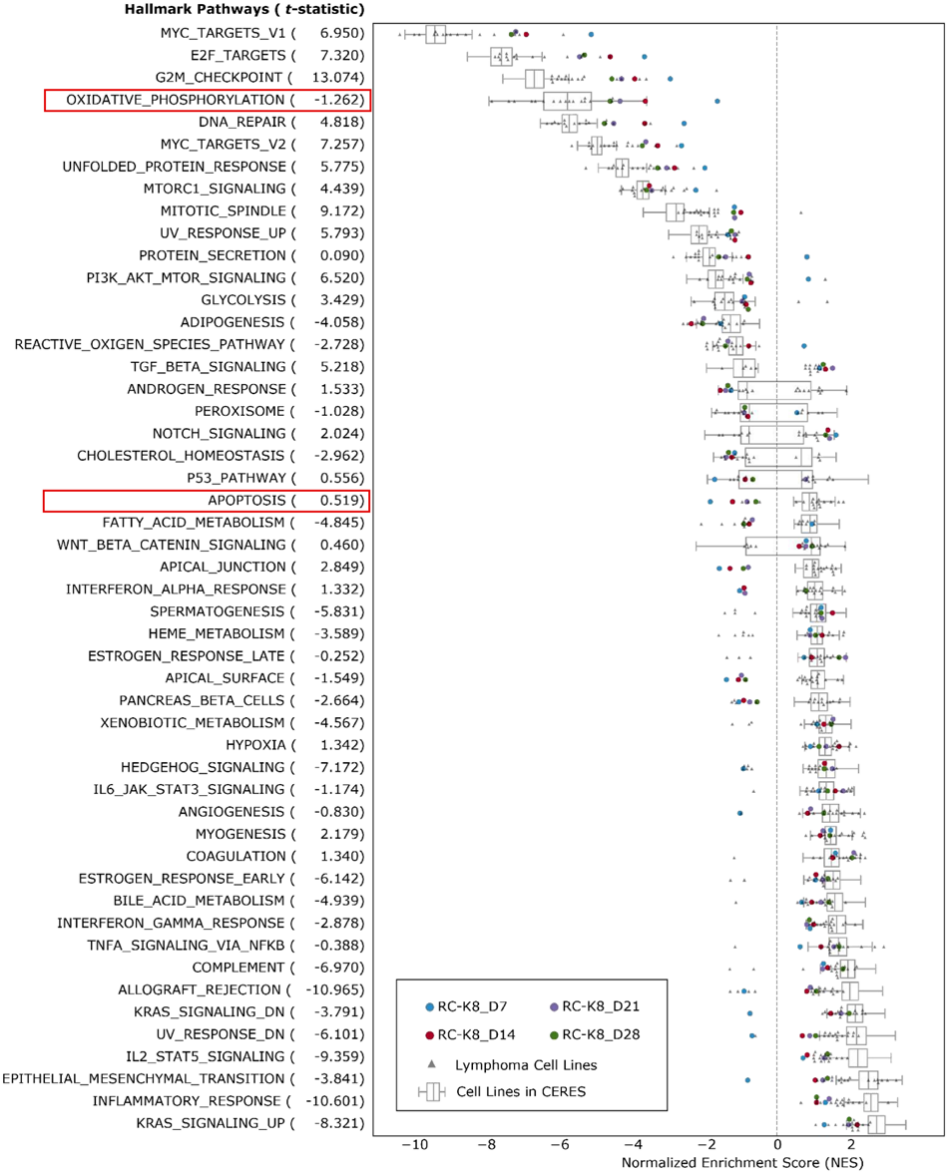
Gene set enrichment analysis (GSEA) of 50 hallmark pathways. Normalized gene enrichment scores based on RC-K8 screening results (CRISPR scores) at four time points (coloured dots) compared to enrichment scores based on previously reported screens from B-cell lymphoma cell lines (grey triangles) and 341 cancer cell lines from the CERES data (box plot). Pathways are sorted by median enrichment scores of all cell lines. T-statistics were calculated by comparing the enrichment scores between lymphoma cell lines and CERES cell lines, indicating the specificity of enrichments towards B-cell lymphoma.

The standardized CRISPR scores at day 7 exhibited a weak correlation (Pearson’s *r* < 0.25) with the average CERES scores (representing general essentialities in 341 cancer cell lines). In contrast, the cumulative effects at later time points correlated more strongly with the CERES scores, with the highest correlation at day 28 (Pearson’s *r* > 0.48) (Supplementary Fig. S5 and Table S1). We surmise that rapid depletions were caused by genetic lethality, whereas delayed dropouts were associated with impaired cellular function or fitness. We hypothesize that essential genes exhibiting different rates of depletion may participate in complementary pathways. To test this hypothesis, based on the ranks of CRISPR scores, we performed gene set enrichment analysis (GSEA) on 50 hallmark pathways (Fig. 4 and Supplementary Table S3). We observed that the most differentially enriched pathways between early and late time points (day 7 vs. day 28) were the apoptosis and oxidative phosphorylation (OXPHOS) pathways (Fig. 4). Compared to normalized enrichment scores (NES) of the 341 cell lines from the CERES data set, the rapid depletion of apoptosis-related genes showed cell-type specificity (Fig. 4), where *BCL2L1*, *CREBBP* and *WEE1* were among the most significant genes (Supplementary Table S1). On the other hand, depletions associated with OXPHOS genes showed a gradual increase over time (Fig. 4), suggesting that growth suppression caused by interrupted energy metabolism is slow and cumulative.

The data also allowed us to investigate, within the same analysis, the relative effects associated with JQ1 (a BET inhibitor) treatment during the screening process (Supplementary Table S4). BET inhibition by JQ1 has been suggested to trigger cell cycle arrest followed by apoptosis or senescence^33^, and we indeed observed a long-term growth-suppressive effect in the JQ1-treated cells. We found no significant enrichment of positively selected genes (those possibly causing resistance to the JQ1 treatment). Nonetheless, several negatively selected genes were found to be associated with the mitogen-activated protein kinase signalling pathway, including *PAK2*, *LAMTOR3* and *MAPKAPK2* (Supplementary Table S1). Notably, the top candidate gene, *PAK2*, was non-essential in RC-K8 cells in general but was selectively depleted in the JQ1-treated cells. This may indicate that the loss of PAK2 could sensitize RC-K8 cells to JQ1 treatment

### DLBCL cells with either *EP300* or *CREBBP* mutations are sensitive to HAT domain

**Fig. 5:**
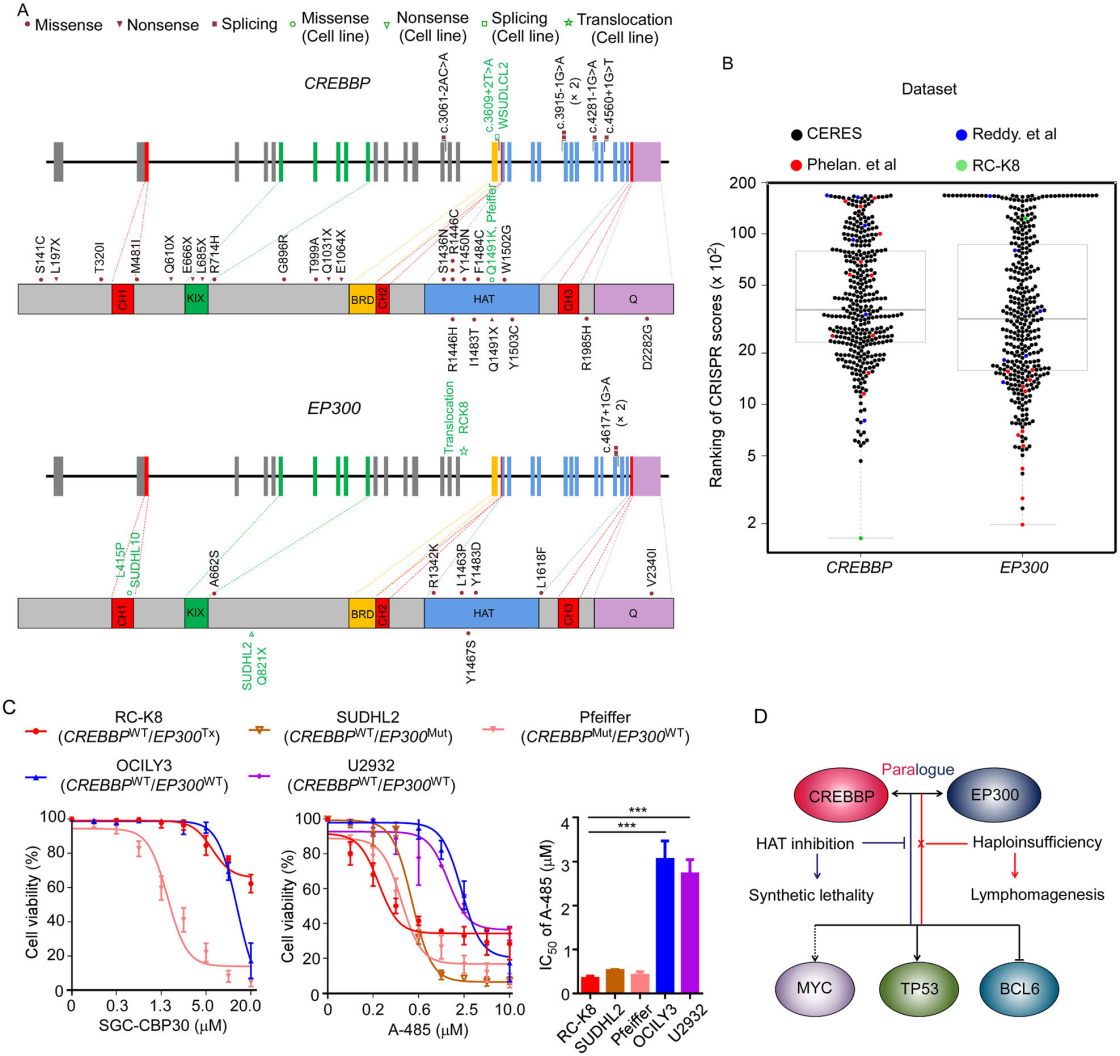
DLBCL cells with genetic alterations in *EP300* and *CREBBP* are sensitive to a HAT domain inhibitor. **A** Somatic mutations in *CREBBP* and *EP300* identified in a previously described DLBCL cohort, RC-K8 cells and other cell lines included by a previous CRISPR screen or tested by A-485 in this study. **B** Ranking of CRISPR scores of *CREBBP* and *EP300* from different datasets. The middle boxes represent the middle 50% of values for each group with a midline as the median value. The bars outside the boxes represent the 1.5 interquartile ranges outside of the boxes. **C** Cell viability tests of targeted inhibition of the BRD domain by the small molecule SGC-CBP30 and the HAT domain by A-485. Error bars: SD (*n* = 3 replicates). The three HAT-mutant cell lines displayed higher sensitivity to A-485 than the cell lines (OCILY3 and U2932) with wild-type HATs, with RC-K8 cells exhibiting the lowest IC_50_ value. Mut: mutation; Tx: translocation; WT: wild-type. **D** Potential mechanism of HAT inhibition in HAT-deficient cells: haploinsufficiency of *CREBBP* or *EP300* promotes lymphomagenesis via dysregulation of BCL6 and TP53; the deficiency develops dependency on the remaining HAT function simultaneously. Targeted inhibition of HAT could cause synthetic lethality through abrogation of MYC.

By re-analysing a previously published DLBCL cohort (275 patients)^7^, we observed that mutations were more frequently identified in *CREBBP* than in *EP300* (11.7% vs. 3.3%, Fig. 5A), which is in agreement with previous studies^17^. Notably, over 70% of these mutations affected the HAT domain (Fig. 5A and Supplementary Table S5). In most of the affected cases, either *CREBBP* or *EP300* was mutated, but we did identify several cases with potentially oncogenic mutations, affecting both the genes (Supplementary Table S5).

Based on re-evaluating and ranking the CRISPR scores of the reference datasets (the CERES data set on 341 cancer cell lines and two previous published screens on DLBCL cell lines)^12,13,32^, *CREBBP* and *EP300* seemed to be essential in a small set of cell lines (Fig. 5B). Among the DLBCL cell lines analysed, *CREBBP* is almost uniquely essential in RC-K8 cells, whereas *EP300* is more frequently identified as an essential gene (Fig. 5B and Supplementary Table S6), which may reflect the different mutational rates in these genes in DLBCL patients. Importantly, two GCB-like DLBCL cell lines that were highly sensitive to *EP300* knockout harboured mutations (WSUDLCL2) or copy number variation (CNV) loss (SUDHL5) in *CREBBP* (Fig. 5B and Supplementary Table S6).

It has previously been reported that BRDs are also critical in sustaining the proliferation of lymphoma cells via epigenetic regulation. Therefore, we tested the *CREBBP*/*EP300*-specific BRD inhibitor SGC-CBP30 in RC-K8 cells and observed a limited effect on proliferation (Fig. 5C), which prompted us to further perform cell viability tests using a newly reported catalytic inhibitor (A-485) that targets the HAT domain of both *CREBBP* and *EP300*^34^. RC-K8 cells responded to A-485 treatment, with the lowest IC50 value (0.42 µM) compared to four other DLBCL cell lines tested (Fig. 5C and Supplementary Table S6). In general, the three cell lines with *CREBBP* and/or *EP300* mutations showed a higher sensitivity than the two wild-type cell lines for these genes (OCILY3 and U2932) (Fig. 5C), suggesting that dependency of the remaining HAT function is a druggable vulnerability in DLBCL cells with either *CREBBP* or *EP300* genetic alterations (Fig. 5D).

## Discussion

In this study, we performed a genome-wide CRISPR-Cas9 loss-of-function screening and identified *CREBBP* as a vulnerability specific to the ABC-like DLBCL cell line RC-K8, which harboured a translocation that disrupts the *EP300* gene. *CREBBP* is one of the most frequently mutated genes in non-Hodgkin lymphoma (NHL) and usually plays a tumour-suppressing role through epigenetic regulation^35^. Such dependency on *CREBBP* can be best explained by a synthetic lethality associated with the loss-of-function of its paralogue *EP300* (Fig. 5D)^21^. As reported in multiple myeloma^36^, the BRD of *CREBBP* is essential for regulating the DLBCL essential gene *IRF4*. Although *IRF4* also exhibited essentiality in our CRISPR screen with RC-K8 cells, the BRD inhibitor CBP30 did not show efficacy. In contrast, A-485, a potent catalytic inhibitor of HAT domains^34^, showed high efficacy in this *EP300*-deficient cell line. The results suggested that the HAT domain of *CREBBP* is linked to essentiality in RC-K8 cells. By re-evaluating the previously published CRISPR-Cas9 screens from a pan-cancer study and sets of DLBCL cell lines, we also identified a genetic essentiality of *EP300* or *CREBBP* gene in a small subset of cancer cell lines, including a few DLBCL cell lines that are dependent on *EP300* and carry *CREBBP* mutation, copy number loss or translocation. We further observed differences in the IC_50_ values of A-485 in five lymphoma cell lines, indicating a potential correlation between *CREBBP/EP300* deficiency and sensitivity to HAT inhibitors. Our results thus suggest that targeting the remaining HAT function may hold therapeutic potential for B-cell lymphomas with deficiency in either *CREBBP* or *EP300*.

The genetic features of the RC-K8 cell line showed some similarity to the BN2 (based on *BCL6* fusions and *NOTCH1* CNV changes)^4^ or the C1 (based on *BCL6* structural variants and mutations of *NOTCH* signalling pathway components) molecular subtype^3^ but also have distinct genetic features. We also noted that it resembled what we have discovered previously in DLBCL samples associated with HBV infection^7^, with translocations in *BCL6*, copy number changes in *NOTCH1* and *CD70*, and mutations in *ZFP36L1*, *SGK1*, *IKZF3*, *TP63* and *TP73*. It has been shown that the HBV protein HBx can directly interact with *CREBBP*/*EP300* and facilitate the recruitment of the complex onto CREB-responsive promoters, upregulating downstream oncogenes^37^. This was supported by the observation that the *CREBBP*-targeted genes were significantly upregulated in HBsAg^+^ tumours compared to HBsAg^−^ tumours (Supplementary Fig. S6). Targeting the HAT functions of *CREBBP/EP300* can thus be a new direction in developing effective treatment for HBV-associated DLBCL patients, who usually have a poor response to the current therapy^7^.

In addition to *CREBBP*, we also identified the cell line-specific essentiality of *MDM2* in RC-K8, which has an increased level of expression of *MDM2* and a largely unaffected *TP53* gene. Accordingly, the screen also showed a strong positive selection of *TP53* in RC-K8 cells. *TP53* is mutated in ~50% of human cancers and is one of most studied tumour suppressors^38^. In other tumours, including the majority of DLBCLs, however, *TP53* is in its wild-type form and targeting the negative regulators of *TP53*, such as *MDM2*, may be a promising approach^39^. A recent CRISPR-Cas9 screen also identified druggable dependencies in *TP53* wild-type Ewing sarcoma, including *MDM2*, *MDM4*, *USP7* and *PPM1D*^40^.

RC-K8 cells have a normal functional TP53, which is capable of triggering DNA damage responses to CRISPR-Cas9 editing during the screening process^41^. Consequently, the calling of essential genes through a depleting effect might be affected by TP53-induced apoptosis or cell cycle arrest. We therefore used a time-course experimental design to distinguish between essentiality and DNA damage response, as cells carrying dispensable knockouts may recover from the perturbation and then grow back. We observed that many such genes exhibited a rapid depleting effect but were not ultimately essential (Supplementary Table S1). During the time course of the CRISPR screen, we also observed a delayed depleting effect of many essential genes and pathways, notably the OXPHOS-related genes. Such delayed essentialities of metabolic pathways are repeatedly observed in CRISPR-based genetic screens^30,42^. One possible reason for the delayed depletion is the poor correlation between mRNA and protein expression, which has been observed in proteomics studies^43^. Thus, highly expressed genes may provide a buffer to CRISPR-induced perturbations and cause false negative results in essentiality calling. The time-course information, analysed by our optimized method, may help to detect such delayed essentialities in cancer cells.

Our unbiased, genome-wide, time course-based CRISPR-Cas9 screen revealed a number of cell line-specific vulnerabilities, such as *CREBBP* and *MDM2*, and a delayed metabolic dependency of OXPHOS genes. By re-analysing the previously published CRISPR-Cas9 screens, we also identified a genetic essentiality of *EP300* in additional DLBCL cell lines. Considering the high mutation rates of *CREBBP* and *EP300* in DLBCL and follicular lymphoma (FL), the two most common types of NHL, and the prognostic value of these two genes in FL (M7-FLIPI)^44^, the findings of our study provide insights for the development of more effective targeted therapies as well as novel combination treatments that may benefit a large group of patients. It is also important to point out that DLBCL is a highly heterozygous disease, and that each tumour carries a unique combination of genetic alterations, affecting multiple functional pathways. Characterization of the “core fitness” gene for cancer cells and DLBCL essential genes might be informative for prioritization of therapeutic targets^11^; however, integration of genetic/transcriptomic data with unbiased functional screening will still be needed to identify the most effective targeted therapy for individual/subgroup of patients.

## Methods

### Cell culture

DLBCL cell lines RC-K8, OCILY3, SUDHL4 and Pfeiffer were purchased from the Leibniz-Institute DSMZ (Braunschweig, Germany) or the American Type Culture Collection (Manassas, USA). A cell line authentication test was performed for the RC-K8 cell line using PCR single-locus technology (Eurofins Genomics, Germany). U2932 was kindly provided by Dr. G. Enblad’s research group (Uppsala University). All cell lines were cultured in RPMI 1640 medium (Invitrogen, Carlsbad, USA) supplemented with 10% fetal bovine serum (Gibco, Invitrogen, Paisley, UK).

### Whole-genome sequencing

DNA from RC-K8 cells was sequenced using the BGI-500 sequencing platform (BGI-Shenzhen, Shenzhen, China). After quality control, 100 bp paired-end clean reads corresponding to 30× sequencing coverage were acquired. Sequence alignment to the reference genome (hg19) and mutation calling were performed by applying the “best-practice” GATK workflow. Mutations were excluded based on the following criteria: (1) a frequency higher than 0.01 in the ExAC (all and Asian), 1000 Genomes (all and Asian) and ESP6500 databases; (2) minor allele frequency < 10% or >90%; and (3) a reference single nucleotide polymorphism ID (rs) number in dbSNP build 147. Structure variations in the RC-K8 cell line were detected using Manta^45^ as described previously^46^ and copy number variations were estimated using Control-FREEC^47^.

### Transcriptome sequencing

Total RNA of RC-K8 was sequenced at BGI-Shenzhen using the Illumina HiSeq 2000 platform. Raw sequencing reads with adaptors, with >10% unknown bases or with >50% low-quality bases in one read, were filtered out. Clean reads were aligned to the reference transcriptome (Hg19) by SOAP2 and SOAPfusion for detecting gene-fusion events. Gene expression levels were reported as reads per exon kilobase per million.

### CRISPR-Cas9 screen with pooled lentiCRISPRv2 library

Following a previously described procedure^48^, a cell line with stably expressed Cas9 (RC-K8-Cas9) was generated and was subsequently transduced by the pooled lentiCRISPRv2 library (multiplicity of infection ~ 0.3). One hundred fifty million cells were collected 24 h after transduction as the baseline and the remaining cells were cultured under 2.5 µg/ml puromycin (Sigma, Darmstadt, Germany) selection for 10 days. At days 3, 7 and 14, 110, 60 and 60 million cells were collected, respectively, whereas 120 million cells were kept in culture after day 14 and treated with either JQ1 (400 nM) (Tocris Bioscience, Abington, UK) or dimethyl sulfoxide (DMSO) for two additional weeks. For each treatment, 60 million cells were collected at two time points: day 21 and day 28. Genomic DNA was extracted from cells collected at different time points using the Blood & Tissue Kit (Qiagen, Hilden, Germany). Guide sequences were PCR-amplified and sequenced at the Broad Institute using the Illumina NextSeq sequencer following a reference protocol^48^. The screening procedure was performed independently in two replicate experiments.

### Preprocessing of CRISPR-Cas9 screen data

A modified preprocessing step derived from the original protocol (count_spacers.py script)^48^ was applied, trimming raw reads in FastQ format by the spacer sequences from both ends (3′: GTTTT and 5′: CGAAACACC) using CutAdapt (v1.8.3 Martin, 2011). One mismatch was allowed in the 5′ sequence (parameter -e 0.12). The trimmed reads with a length of 18–21 bp were mapped to the FASTA library of guide sequences (GeCKO v1/v2, Addgene.org) using Bowtie2^49^. To increase the specificity of read-count data, we mapped the library against the human genome sequence and excluded guide sequences targeting non-protein coding regions (named with “mir-” or “let-” tags) or having alternative alignments (filtered by “XS” and “NM” tags in the bowtie2 result). After filtering, read-count data were normalized by the median count of each individual experiment.

### Gene essentiality estimation

The MAGeCK maximum-likelihood estimation (MLE) algorithm was used to estimate the relative screening effects (β-scores or modified log fold changes) in both negative and positive directions, i.e., depletion and enrichment. In the design matrix of the MAGeCK analysis, samples were binary coded in different groups based on time points, batches and drug treatments (Supplementary Table S4). Samples from day 1 were labelled the baseline (zero) in all grouping conditions. As a result, one β-score was calculated by MLE for each gene in each condition. To resolve the CRISPR screening effect with the time course, β-scores were fit by linear interpolation curves along the time axis (*k* days). For every gene, given that day 1 is the baseline: *β*(1) = 0, the integral of β-scores at a time point (*k*) was used as the proxy of the overall CRISPR screening effect: $S_k = {\int}_1^k {\beta (t)dt}$. To normalize the integral scores, null distributions of scores were estimated based on a null set of read-count data generated by permutating the identities of sgRNA sequences. Null scores were calculated by MAGeCK-MLE following the same procedure and then fitted by Gaussian distributions at each time point. Subsequently, the original integral scores were standardized by the mean and SD of the null distribution: $Z_{\mathrm{gene} \cdot k} = \frac{{S_{\mathrm{gene} \cdot k} - \bar S_{\mathrm{null} \cdot k}}}{{\sigma \left( {S_{\mathrm{null} \cdot k}} \right)}}$.

To test the validity of the scoring method, two reference datasets (HT-29^30^ and CERES^32^) were used for benchmarking, i.e., the HT-29 data set (colon cancer cell line, with multiple time points, day 3 to day 25) and the CERES data set (341 cancer cell lines, with 1 time point). The Pearson’s correlation coefficients between the estimated CRISPR effects and the general essentialities of the average CERES scores served as the figure-of-merit for overall performance at each time point. In addition, two previously published CRISPR screens were used to characterize essential DLBCL genes^12,13^. To compare the results from different datasets, for each cell line, we also ranked the CRISPR scores for 16,821 genes that were studied in all samples.

GSEA was performed by using the pre-ranked GSEA approach^50^. The 50-hallmark gene sets were used in GSEA, while weights were set to zero (classic method)^51^. One thousand permutations were used and NES were used for comparisons between datasets.

### Single-gene knockout experiments

Two to three sgRNAs for each of the targeted genes and nontargeted controls were chosen from the lentiCRISPRv2 library and individually cloned into the plasmid backbone of the sgRNA library. Lentiviruses were subsequently produced in 293T cells for each selected sgRNA. In each single-gene knockout experiment, three million RC-K8-Cas9 cells were transduced and then selected by puromycin (2.5 µg/ml). The growth of cells was monitored by counting the numbers of living cells at five time points (days 1, 3, 5, 9 and 14). DNA was extracted from cells collected at day 5 to verify the desired targeting (introduction of loss-of-function insertions or deletions) of each selected sgRNA by SURVEYOR assay (Integrated DNA Technologies, Coralville, USA) and sequencing^48^.

### Cell viability assay

A total of 2 × 10^4^ cells were seeded in a volume of 100 μL per well with vehicle or the indicated concentrations of drugs (nutlin-3, Santa Cruz Biotechnology, USA; SGC-30, Sigma-Aldrich; A-485, Selleckchem). All drugs were dissolved in DMSO. At 72 h post treatment, 20 μl per well of CellTiter 96 AQueous One Solution Reagent (Promega, Madison, USA) was added. After incubation of the plates for 4 h at 37 °C, cell viability was measured by comparing the absorbance (A) at 450 nm: *A*_treatment_/*A*_control_ × 100%. Each experiment was independently repeated at least three times.

### Statistics

*P*-values were calculated by Student’s *t*-test/analysis of variance for quantitative comparisons and *χ*^2^ or Fisher’s exact test for categorical comparisons.

### Data sharing statement

The CRISPR scores and read counts for gRNA for the CRISPR screen are shown in Supplementary Tables S1 and S7, respectively. The WGS and RNA-seq data that support the findings of this study have been deposited into CNGB Sequence Archive of CNGBdb^52^ with accession number CNP0001595.

## Supplementary information

Figure S1

Figure S2

Figure S3

Figure S4

Figure S5

Figure S6

Supplementary figure legend

Table S1

Table S2

Table S3

Table S4

Table S5

Table S6

Table S7
