# Supplementary figures and images for: Genome-wide CRISPR screens reveal synthetic lethal interaction between CREBBP and EP300 in diffuse large B-cell lymphoma
Source: Cell Death Dis. 2021 Apr 28;12(5):419. doi: 10.1038/s41419-021-03695-8 (PMC8080727; doi:10.1038/s41419-021-03695-8)

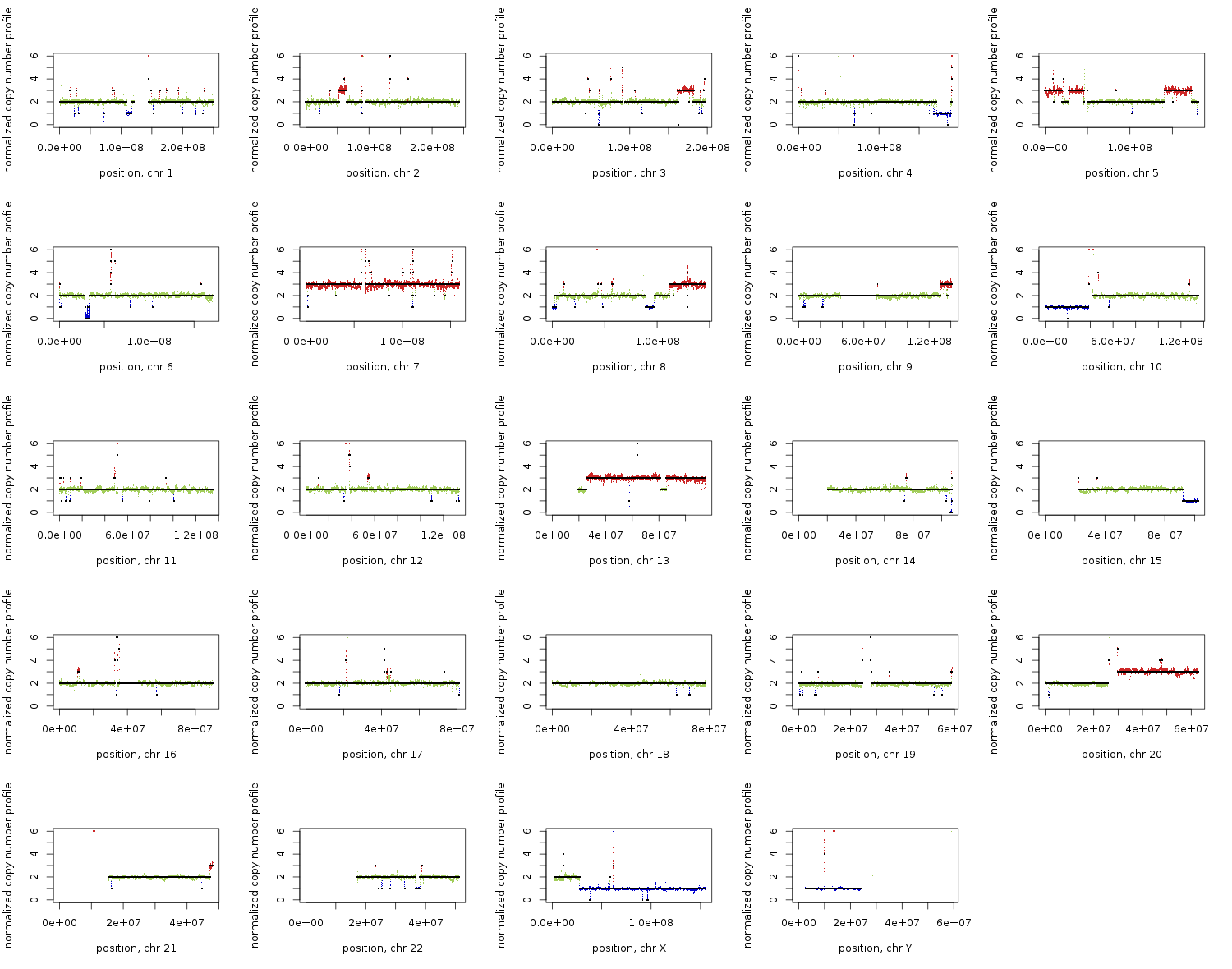

Supplement: Supplementary file 1 — Figure S1 [file 41419_2021_3695_MOESM1_ESM.png]

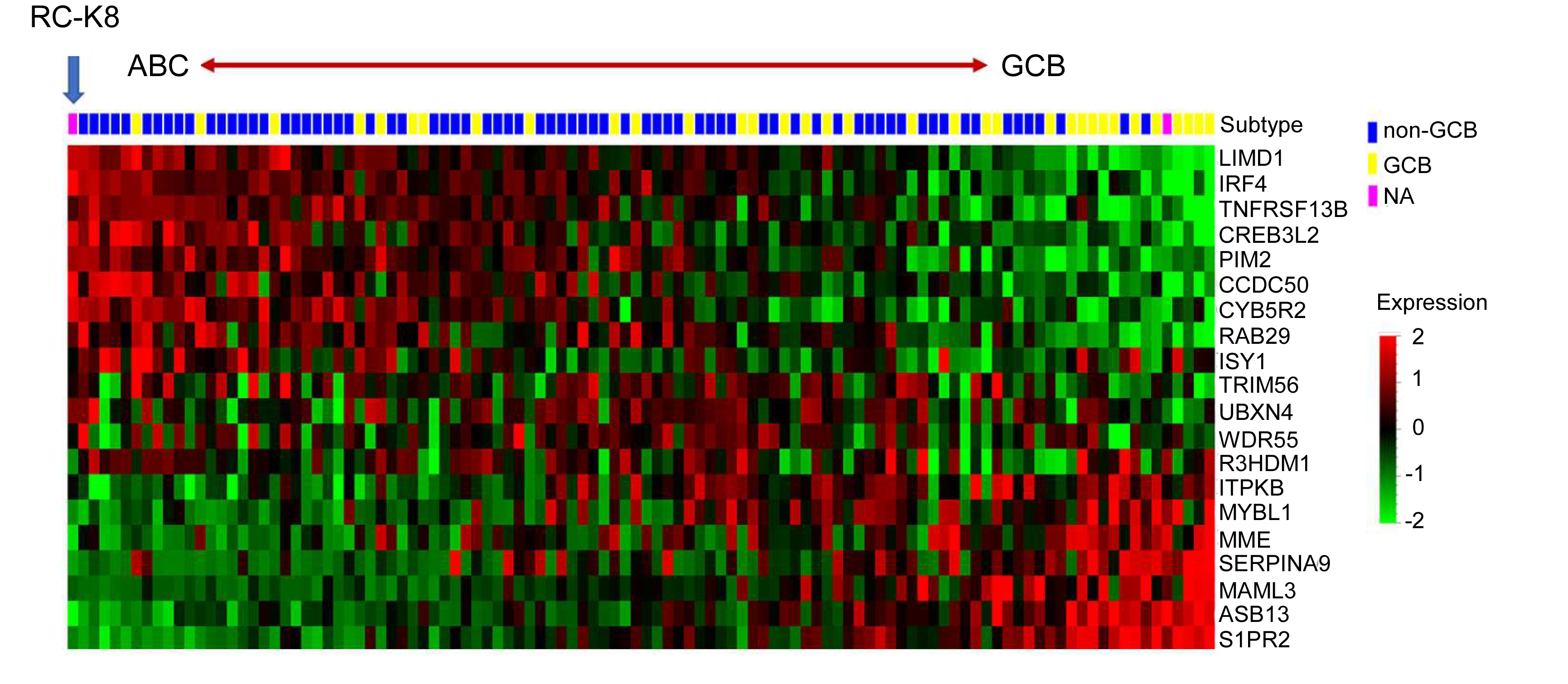

Supplement: Supplementary file 2 — Figure S2 [file 41419_2021_3695_MOESM2_ESM.tif]

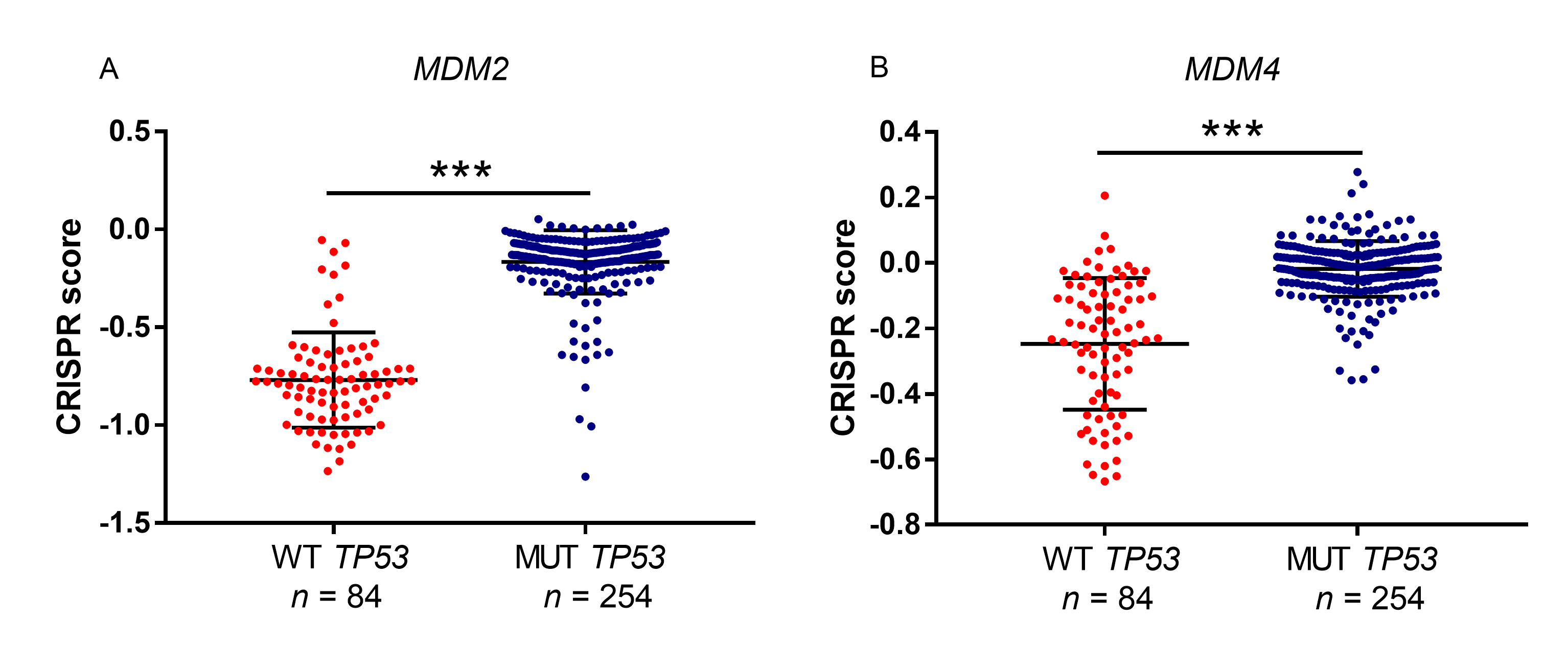

Supplement: Supplementary file 3 — Figure S3 [file 41419_2021_3695_MOESM3_ESM.tif]

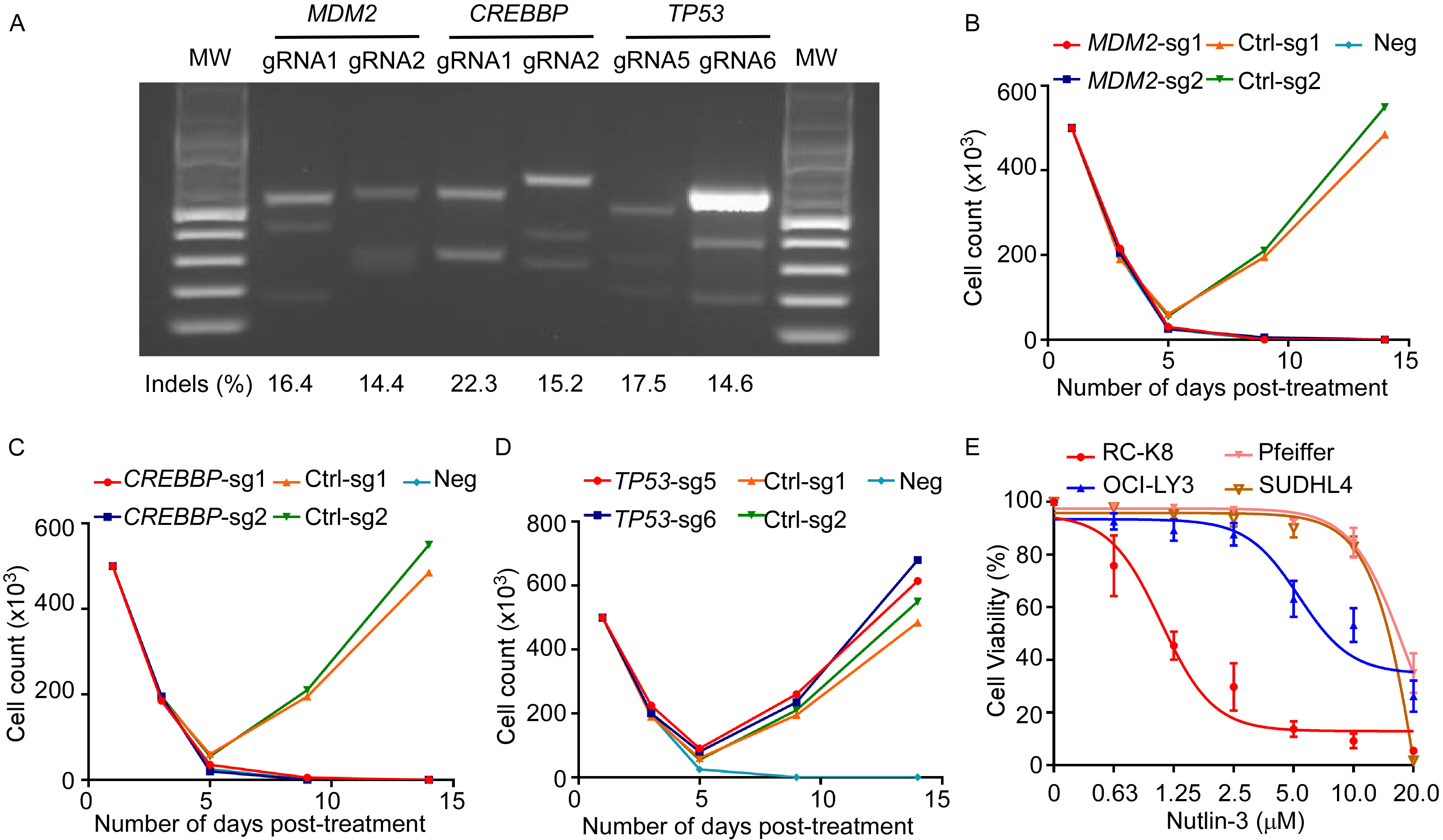

Supplement: Supplementary file 4 — Figure S4 [file 41419_2021_3695_MOESM4_ESM.tif]

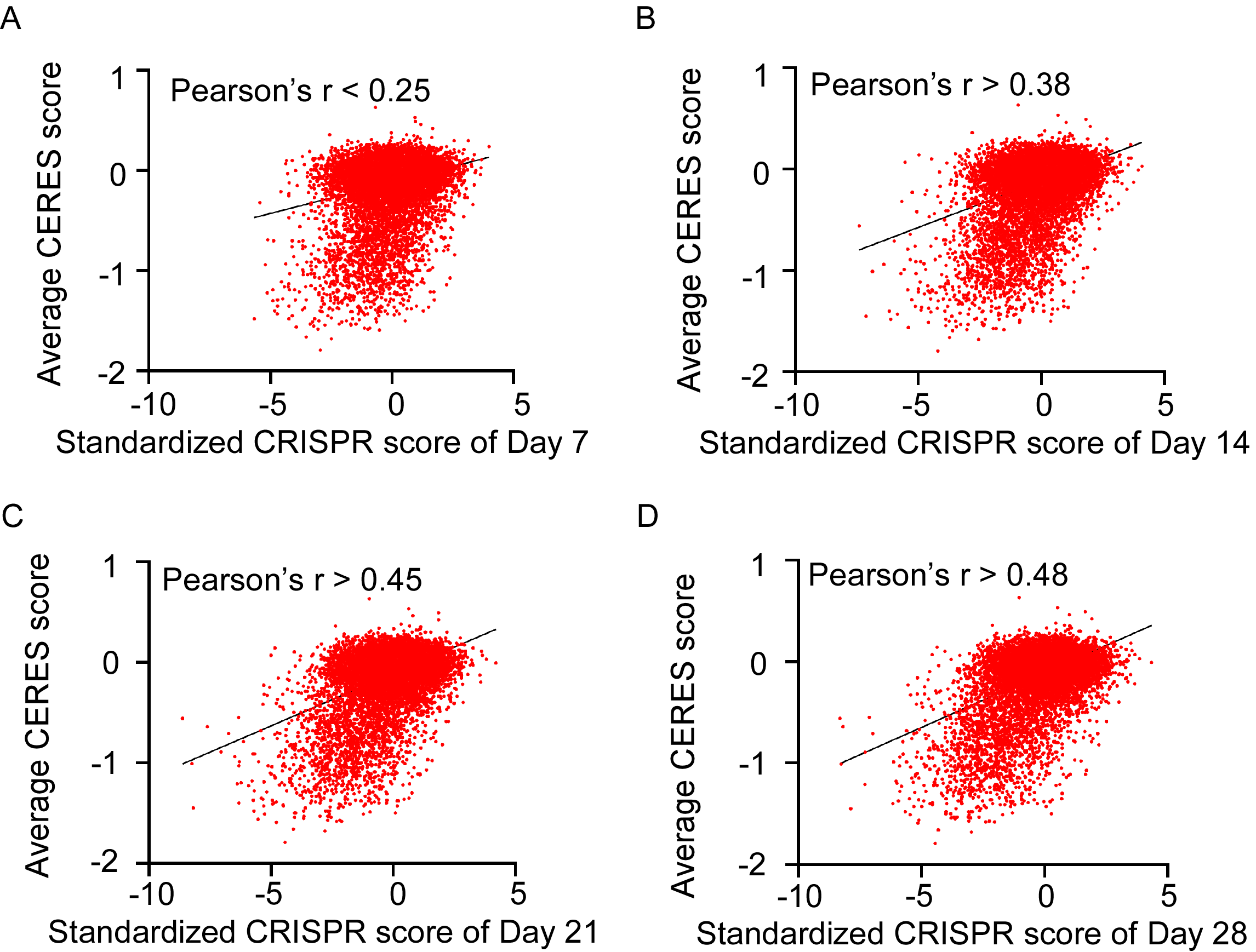

Supplement: Supplementary file 5 — Figure S5 [file 41419_2021_3695_MOESM5_ESM.tif]

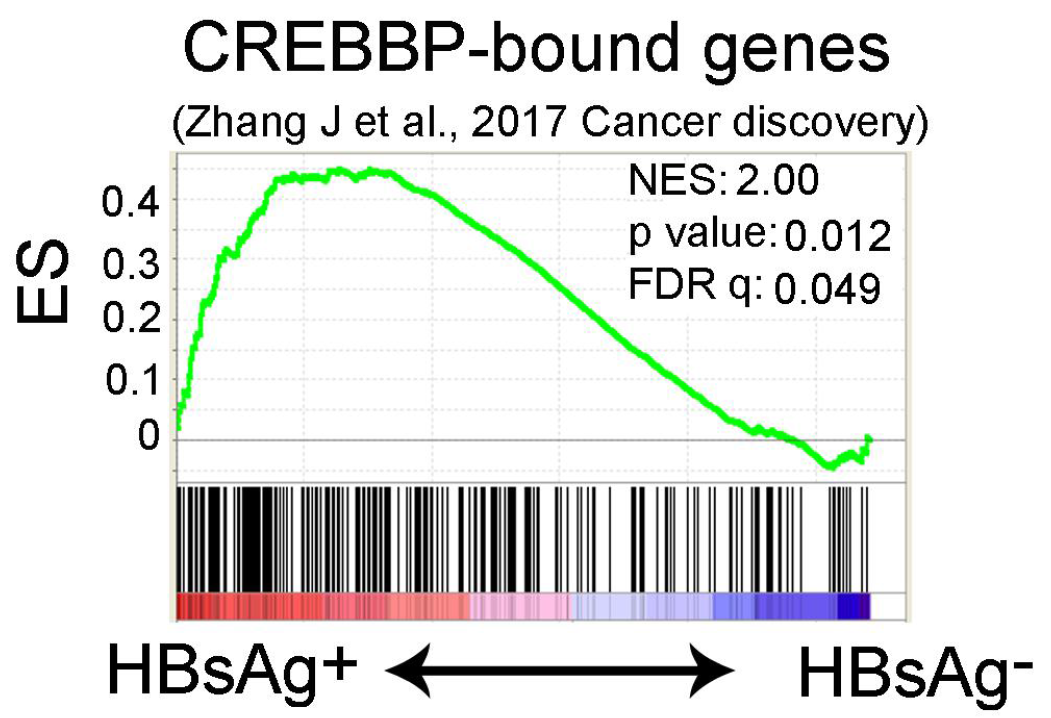

Supplement: Supplementary file 6 — Figure S6 [file 41419_2021_3695_MOESM6_ESM.tif]
