# Supplementary material for: Genome-wide CRISPR screens reveal synthetic lethal interaction between CREBBP and EP300 in diffuse large B-cell lymphoma
Source: Cell Death Dis. 2021 Apr 28;12(5):419. doi: 10.1038/s41419-021-03695-8 (PMC8080727; doi:10.1038/s41419-021-03695-8)
Supplement: Supplementary file 7 — Supplementary figure legend [file 41419_2021_3695_MOESM7_ESM.docx]

**Supplementary information**

**Genome-wide CRISPR screens reveal synthetic lethal interaction between CREBBP and EP300 in diffuse large B-cell lymphoma**

Man Nie, Likun Du, Weicheng Ren, Julia Joung, Xiaofei Ye, Xi Shi, Sibel Ciftci, Dongbing Liu, Kui Wu, Feng Zhang, Qiang Pan-Hammarström

**Supplementary figure legends**

**Figure S1 | Overview of copy number variations (CNVs) in RC-K8 cell line.** CNVs of genomic regions were analysed by using the WGS data and the Control-FREEC tool. Whole chromosome or large part of chromosome gains of chromosomes 7, 5, 13 and 20 were observed. Detailed information for CNVs at gene level is shown in Table S1.

**Figure S2 | Gene expression profile revealed an ABC-like feature of the RC-K8 cell line.**

**Figure S3| *MDM2* and *MDM4* were more essential in cancer cell lines with WT *TP53*.** In CERES dataset, CRISPR score of *MDM2* (A) and *MDM4* (B) were significantly lower in cancer cell lines with WT *TP53* (*n* = 84) than MUT *TP53* (*n* = 254). Student’s *t* test, ****P* < 0.001.

**Figure S4 | Validation of top candidates.** (**A**) SURVEYOR assay was used to determine the indel ratio of transfected RC-K8 cells. Cas9-mediated cleavage efficiency (indel ratio) was calculated on the basis of integrated intensity of gel binds. Genomic DNA was extracted from cells on day 5 post transduction. (**B-D**) RC-K8 cells transduced with *MDM2*-targeting lentiCRISPRs (**B**) and *CREBBP*-targeting lentiCRISPRs (**C**) showed significant growth inhibition, whereas cells transduced with lentiCRISPR vectors did not. RC-K8 cells transduced with *TP53*-targeting lentiCRISPRs (**D**) showed no changes in growth compared to cells transduced with lentiCRISPR vectors; these experiments were run in parallel with the same negative control and control gRNAs. (**E**) Cells were treated with MDM2 inhibition by the small molecule nutlin-3 for 72 h. The data represent the mean ± SD of three independent experiments.

**Figure S5|** Scatter plots showing the correlation between the standardized CRISPR scores of RC-K8 at different time points and the average CERES scores. Pearson correlation values are indicated.

**Figure S6 | CREBBP-bound gene set was significantly upregulated in HBsAg^+^ DLBCLs.** GSEA plot illustrating the enrichment of CREBBP-bound genes in HBsAg^+^ DLBCLs compared to HBsAg^-^ DLBCLs. RNAseq data was described previously and re-analyzed here. GSEA were performed with 1000 sample permutations. Enrichments were considered significant if FDR q < 0.25. ES, enrichment score. NES: normalized enrichment score.
